# Front-Line Treatment of High Grade B Cell Non-Hodgkin Lymphoma

**DOI:** 10.1007/s11899-019-00518-8

**Published:** 2019-06-29

**Authors:** Murali Kesavan, Toby A. Eyre, Graham P. Collins

**Affiliations:** 1 Department of Clinical Haematology, Oxford Cancer and Haematology Centre, Churchill Hospital, Oxford University Hospitals NHS Trust, Oxford, OX3 7LE UK; 2 University of Oxford Department of Oncology Clinical Trials Unit, Churchill Hospital, Oxford University Hospitals NHS Trust, Oxford, UK

**Keywords:** Chemoimmunotherapy, Diffuse large B cell lymphoma, Primary mediastinal B cell lymphoma, Elderly, Novel agents, Dose intensity

## Abstract

**Purpose of Review:**

Rituximab-based chemoimmunotherapy has resulted in a marked improvement in the survival of diffuse large B cell lymphoma (DLBCL). We reflect upon the history front-line (1L) therapy and highlight advances in management.

**Recent Findings:**

Since the introduction of R-CHOP, the majority of randomized studies in the front-line treatment of DLBCL have failed to show a benefit. Such studies have involved treatment intensification, adding novel agents to the R-CHOP backbone and targeting such novel agents to biologically defined subgroups. R-CHOP therefore remains standard-of-care for most but new insights into the molecular biology of these diseases, and the development of active targeted molecules offers promise for the future. Accumulating evidence in the very elderly suggests dose attenuation does not compromise survival. Intensification in primary mediastinal B cell lymphoma may avoid the need for radiotherapy, but must be balanced against the risks. PET-CT- and ctDNA-based response assessment may now enable response adapted therapy and early prognostication, improving patient selection and potentially outcomes.

**Summary:**

Novel technologies and therapies in combination with novel molecular diagnostics will likely become the standard-of-care approach for the personalized therapy of DLBCL but need to be proven in well-designed and conducted randomized trials.

## Introduction

Diffuse large B cell lymphoma (DLBCL) accounts for 30–40% of non-Hodgkin lymphoma (NHL) [1] and is more common in older patients with a median age of diagnosis of 70 years [2]. Numerous variants are classified by the World Health Organization (WHO) and are often defined by anatomical site of involvement [3]. Historically considered a DLBCL subtype, primary mediastinal B cell lymphoma (PMBCL) represents 2–4% of NHL [4]; however, with the availability of gene expression profiling (GEP), it is now recognized as a separate entity. Despite molecular analysis, the largest subgroup remains ‘not otherwise specified’ (DLBCL-NOS). Further analysis is rapidly unravelling our understanding of pathobiology of these entities and revealing potential targets for therapy. Adoption of first-line (1L) chemoimmunotherapy over the last 15 years has improved outcomes for many DLBCL and PMBCL patients. However, a significant minority fail to respond or relapse early after 1L. Herein, we discuss 1L DLBCL therapy, focusing on DLBCL-NOS, double-hit lymphoma (DHL) and PMBCL. Due to an increased incidence in the elderly, we will also focus on this challenging population.

## DLBCL-NOS

Within DLBCL-NOS, the germinal centre B cell subtype (GCB) and activated B cell subtype (ABC) are now defined as ‘cell of origin’ (COO) subtypes according to molecular features (2016 WHO). These were first identified using gene expression profiling (GEP) [5] and although attempts have been made to use immunohistochemistry (IHC) [6, 7], GEP remains the gold standard. Initial reports suggested inferior outcomes for ABC subtypes both in rituximab-naïve and exposed patients [5, 8, 9]. Furthermore, the role of MYC in terms of the impact on DLBCL prognosis has become better defined. Co-expression of MYC and BCL2 protein as demonstrated by IHC predicts for an inferior outcome for patients treated with R-CHOP (rituximab, cyclophosphamide, vincristine, prednisolone) [10, 11]. *MYC* alongside *BCL2* and/or *BCL6* gene translocation (so-called ‘double’ or ‘triple hit lymphoma’ (DHL or THL)) defined by fluorescence in situ hybridization (FISH) is uncommon but associated with a significantly inferior prognosis [10, 12].

### Challenging R-CHOP by Dose Intensification

R-CHOP is considered the standard-of-care (SOC) for DLBCL-NOS irrespective of the COO. In the pre-rituximab era, CHOP became the established chemotherapy backbone following a randomized trial that compared it with numerous intensive multi-agent regimens. This study demonstrated equivalent efficacy and a superior toxicity profile [13]. Rituximab improved outcomes with little additional toxicity, in high- and low-risk patients [14, 15]. Until recently, the ideal delivery of the R-CHOP schedule was controversial. The German group established dose-dense CHOP given every 14 days (CHOP-14), requiring granulocyte colony stimulating factor (G-CSF) support as their standard regimen. With the efficacy of rituximab established, R-CHOP-14 was compared with R-CHOP-21 (every 21 days). R-CHOP-14 failed to show a benefit [16, 17]. R-CHOP-21 was therefore considered the standard approach.

Follow-up in vitro studies suggested that continuous drug exposure increased cell kill compared with more limited bolus exposure [18, 19]. The US National Cancer Institute (NCI) investigated a 96-h continuous infusion of vincristine, doxorubicin and etoposide which forms part of the EPOCH regimen (other components: prednisolone, cyclophosphamide). Serial blood count monitoring is a critical component of this regimen, with dose escalation in subsequent cycles if little or no myelosuppression is observed. Myelosuppression acts as a surrogate pharmacodynamic marker, indicating that a therapeutic steady state of infusional drugs has likely been reached. An initial phase 2 study of dose-adjusted EPOCH with rituximab (DA-EPOCH-R) showed a complete response/unconfirmed complete response (CR/CRu) rate of 94% with a 5-year progression-free survival (PFS) rate of 79%, suggesting high activity [20]. A CALGB/Alliance randomized phase III trial was performed in newly diagnosed stage II-IV DLBCL patients comparing R-CHOP with DA-EPOCH-R. The trial took 8 years to recruit 524 patients. At a median follow-up of 5.2 years, there was no difference in the primary endpoint of PFS (5-year PFS 66% (R-CHOP) versus (vs.) 68% (DA-EPOCH-R)) [21]. Further, despite the lower than expected incidence of the MYC rearrangements and double expressor phenotypes (BCL-2 ≥ 50% and MYC ≥ 40% by IHC), no significant difference by treatment arm was demonstrated in either of these higher risk groups. However, the toxicity of DA-EPOCH-R was significantly higher than R-CHOP. For example, grade (G) 3-4 neutropenic fever (NF) was observed in 90% and 56% respectively and G3-4 sensory neuropathy in 15% and 3% respectively. Further, practical delivery is more cumbersome, so R-CHOP has remained standard [21•, 22].

An important question is whether dose-dense regimens may benefit higher risk DLBCL subtypes. DA-EPOCH-R in *MYC*-rearranged DLBCL has been investigated in a phase II trial of 53 patients (19 isolated *MYC* rearrangement; 24 *MYC* and *BCL2* and/or *BCL6* rearrangement). After a median follow-up of 55.6 months, the 48-month event-free survival (EFS) was 71% with 3 treatment-related deaths [23]. Retrospective analyses also support intensive therapy such as DA-EPOCH-R, or R-HyperCVAD/MA (rituximab, cyclophosphamide, vincristine, doxorubicin, dexamethasone alternating with high-dose methotrexate and cytarabine) in this setting [24, 25]. A meta-analysis, including 11 retrospective studies and 394 patients, concluded that dose intensive therapy improves PFS but not overall survival (OS) [26]. The retrospective nature of these studies makes it impossible to control for confounding factors such as fitness and comorbidities. It is unsurprising that some centres have consolidated induction chemoimmunotherapy with stem-cell transplantation for DHL. Although data is sparse, generally the literature does not support such an approach, especially if dose intensive induction is used [24, 27].

Fractionated cyclophosphamide, vincristine, doxorubicin, methotrexate alternating with ifosfamide, etoposide, cytarabine (CODOX-M/IVAC) is an alternating dose-dense regimen originally developed for Burkitt lymphoma (BL). Alongside rituximab, CODOX-M/IVAC was investigated in a single arm phase II study in international prognostic index [28](IPI) 3-5 DLBCL patients (BL was included but analysed separately). One hundred sixteen truly high-risk DLBCL or high grade B cell lymphoma patients (9% CNS involvement and 53% performance status (PS) ≥ 2) with a median age of 50 years (range 18–65) were treated [29]. At a median follow-up of 53 months, the 3-year PFS and OS were 68.4% and 76.2% respectively. The treatment-related mortality (TRM) was 4.3% with all deaths in patients > 50 years and PS 3. The major issue here, as with other studies, is the non-randomized single arm design, which introduces significant uncertainties. A randomized study is required to increase certainty that exposure to more intensive, toxic regimens is justified in higher risk patients.

The French have compared a dose intense regimen R-ACVBP (rituximab, doxorubicin, cyclophosphamide, vindesine, bleomycin, prednisolone) induction and subsequent consolidation (high-dose methotrexate, ifosfamide, etoposide and cytarabine), with 8 cycles of R-CHOP in a randomized phase III trial [30]. Relatively low risk patients with an age-adjusted IPI of 1 were recruited. After a median follow-up of 44 months, the 3-year EFS was 81% (R-ACVBP) vs. 67% (R-CHOP). Furthermore, an OS difference was evident, 92% for R-ACVBP vs 84% for R-CHOP. Outside of France, however, this regimen has not been widely adopted. The outcome for the R-CHOP control arm was worse that might be expected, the toxicity of R-ACVBP was considerably higher than with R-CHOP, and the relevance to high IPI patients was uncertain. Therefore, R-CHOP-21 remains standard.

### Introducing Novel Agents Into the R-CHOP Backbone

Recent trials have assessed the addition of a novel agent alongside R-CHOP. This has frequently been guided by preceding biological studies, some of which have predicted subtypes most likely to benefit from their addition. For example, the Bruton’s Tyrosine Kinase (BTK) inhibitor ibrutinib, proven so active in indolent B cell disorders, was investigated in relapsed/refractory (R/R) DLBCL. Mutation analysis of cases with ABC-GEP suggested this subgroup is more likely to respond due to the more frequent presence of B cell receptor (BCR) pathway activating mutations and chronic active BCR signalling [31]. Studies in patients confirmed an enrichment of responses in those with ABC-GEP [32]. In a phase II study, 80 patients with R/R DLBCL, a 37% response rate was seen in patients with an ABC subtype compared with 5% with GCB treated with ibrutinib monotherapy. However, responses were short-lived in both subgroups; the median response duration was 4.8 months in the ABC group. These findings led to a large randomized, placebo-controlled, double-blind phase III study comparing 6-8 courses of R-CHOP alongside ibrutinib, with 6-8 R-CHOP in the 1L setting [33•]. Eligibility was restricted to ABC subtype determined by IHC despite the gold standard being RNA-based GEP [34]. The so-called PHOENIX trial randomized 838 patients with a median age of 62 years. At a median follow-up of 34.8 months, no difference in EFS was noted (hazard ratio (HR) 0.93 (confidence interval (CI) 0.73–1.20). Interestingly, a pre-planned subgroup analysis showed a significant interaction with age, with those < 60 years showing a more favourable outcome than those > 60 years. This was due to an increase in toxicity of the combination in patients aged > 60 years resulting in a significantly higher discontinuation rate and lower dose intensity for those on the experimental arm. In those aged < 60 years, a significant improvement in EFS, PFS and OS was observed in the experimental arm. However, as the trial was not designed or powered to assess outcomes by the age group alone, it is not possible to recommend a change in the SOC. Further investigation of these agents in 1L setting of DLBCL may be warranted.

Bortezomib is a proteasome inhibitor which has a complex mechanism of action thought to involve the NF-κB pathway [35]. This would predict preferential activity in the ABC subtype as this is characterized by more frequent activation of the NF-κB pathway [36]. The UK NCRI group tested the incorporation of bortezomib into the 1L setting. In the REMoDL-B randomized phase III clinical trial, all enrolled patients with previously untreated DLBCL received an initial course of R-CHOP during which time formalin-fixed biopsy material was sent for RNA GEP [37•]. The result then enabled randomisation stratified according to GEP-defined COO, from course 2 onwards, between R-CHOP and R-CHOP plus bortezomib. 1128 patients were registered with 918 being randomized. There was an enrichment for GCB-COO (51.7% of patients with biopsy material available), and 26.6% had ABC subtype with 21.7% unclassifiable. There was no difference in the primary endpoint of PFS in the ABC and GCB jointly analysed group or indeed when analysed separately. However, this study provides proof-of-principle that, given sufficient biopsy material is available, GEP can be performed in a time frame relevant for making treatment decision early in 1L.

There are number of ongoing phase III studies investigating novel agents alongside R-CHOP. The ROBUST study (NCT02285062) is a randomized, double-blind, placebo-controlled phase III study assessing R-CHOP plus lenalidomide 10 mg o.d. on days 1–10/21-day cycle (R^2^-CHOP) versus R-CHOP in ABC subtype DLBCL assessed by GEP conducted screening [38]. Nowakowski and colleagues had initially reported on 64 patients with newly diagnosed DLBCL patients treated with R^2^-CHOP and compared them with a contemporaneous R-CHOP-treated case-matched control population. As expected, the control group had worse survival in patients with ABC subtype versus GCB subtype. However, there was no difference seen in R^2^-CHOP patients suggesting a selective efficacy benefit in the ABC subtype [39]. The multicentre FIL REAL07 study assessed 49 patients with newly diagnosed DLBCL or grade 3b follicular lymphoma with R^2^-CHOP (lenalidomide 15 mg o.d. on days 1–14/21-day cycle). Again, outcomes in GCB and non-GCB (determined by IHC) were equivalent [40]. Despite the promise of these earlier trials, Celgene recently announced that the ROBUST study has not met its primary endpoint of demonstrating superiority in PFS compared with placebo plus R-CHOP [41]. This result highlights the importance of prospective randomized trials in the era of novel agent combinations. However, in view of the inferior ROBUST trial outcomes, results of the similar ECOG/ACRIN randomized phase II study of R^2^-CHOP versus CHOP for newly diagnosed DLBCL (NCT01856192) are eagerly anticipated. The POLARIX study (NCT03274492) is another randomized, placebo-controlled, double-blind phase III trial, in newly diagnosed DLBCL patients with IPI ≥ 2. R-CHOP is compared with R-CHP alongside Polatuzumab vedotin (Pola), the anti-CD79b antibody-drug conjugate (ADC) with the tubulin inhibitor toxin monomethyl auristatin E (MMAE). The drug has shown interesting activity in R/R DLBCL. A small randomized trial has compared bendamustine and rituximab (BR) with BR-Pola [42]. The response rate was 70% (BR-Pola) compared with 33% (BR). Although underpowered, the median PFS was also significantly prolonged (6.7 vs. 2 months) as was the median OS (11.8 vs. 4.7 months). It will be interesting to see if this signal of activity in R/R disease translates to benefit in 1L setting.

## Better Defining High-Risk DLBCL

As discussed, many strategies to improve front-line treatment of DLBCL have targeted high-risk patients although this has typically been based on the IPI or COO. It is accepted that very high-risk patients are those with *MYC* rearrangements combined with rearrangements of *BCL2* and/or *BCL6*. However, they represent a small proportion. Recently, further molecular analysis has expanded our understanding of very high-risk DLBCL. The Vancouver group investigated the GEP of patients with DHL and defined a 104-gene ‘double-hit signature’ (DHITsig). This signature classified 27% as having a double-hit profile even though only half actually harboured translocations which define DHL [43]. In a validation cohort, the 5-year OS was 76% in DHITsig-negative GCB patients compared with only 46% in DHITsig-positive GCB cases. In a similar study using material from REMODL-B, a GEP signature previously identified to be BL-like was applied [44]. Nine percent harboured the so-called ‘Molecular High Grade’ (MHG) signature with 90% found within the GCB subtype. The 3-year PFS was 37% in the MHG group compared with 78% for MHG-negative GCB cases and 64% for the MHG-negative ABC subtype. The MHG encompassed most patients with DHL but importantly expanded the high-risk group to more than double the number of DHL cases. Both studies came to similar conclusions, identifying an enlarged group of very high-risk patients enabling trials to target this population.

Rather than identifying very high-risk patients pre-1L, an alternative approach is the early identification of non-responders during 1L. The PETAL study enrolled 862 patients with high grade NHL (majority DLBCL) and treated all with initially 2 cycles of CHOP (adding rituximab for B cell lymphoma) [45]. An interim ^18^F-fluorodeoxyglucose labelled positron emission tomography/computer tomography scan (PET-CT) was performed at least 10 days post-cycle 2 (R)-CHOP (preferably 21 days). No G-CSF was used in cycle two to avoid interference with PET-CT interpretation. A negative PET-CT was defined as a reduction in the Standardized Uptake Value (SUV) > 66% with a positive scan failing to meet this SUV reduction. A positive interim PET-CT was observed in 12.5% and was associated with a poor EFS of < 50% irrespective of IPI (also true for the DLBCL subgroup). The trial involved a randomisation for PET-CT positive patients to continue up to 8 cycles of (R)-CHOP or switching to an intensive ‘BL-treatment’ approach. No benefit was seen by intensifying treatment but more toxicity was observed. This further underscores the conclusion that intensifying chemotherapy is not necessarily better in DLBCL when assessed in randomized trials, even in high-risk patients.

A recent innovation is the detection of circulating, cell-free tumour-associated DNA (ctDNA) in DLBCL [46, 47]. ctDNA with disease-specific somatic mutations was found in 98% of 217 patients with DLBCL pre-treatment [48]. A 2-log reduction, termed ‘early molecular response’ (EMR), was seen in most patients post-1 cycle of R-CHOP, and a 2.5 log reduction termed a Major Molecular Response (MMR), post-2 cycles. In validation sets, failure to achieve EMR or MMR was associated with a significantly worse EFS, with a 3-year PFS for EMR/MMR positive of 83% and 82% respectively, compared with a 3-year PFS for EMR-negative/MMR-negative of 50% and 46% respectively. These values remained independent prognostic markers on multivariable analysis which included IPI variables and interim PET results.

Our ability to identify very high-risk patients at diagnosis and early during 1L is therefore rapidly improving. Currently, however, there is no evidence in support of a risk-adaptation strategy that can be relied upon to guide treatment in high-risk individuals. Due to the failure of more intensive chemoimmunotherapy seen in PETAL, alternative approaches should be investigated early in the treatment algorithm such as Chimeric Antigen Receptor T cell (CAR-T cell) therapy, which has proven efficacious in R/R DLBCL [49, 50].

## Management of Elderly Patients

The incidence of DLBCL in the elderly is disproportionally growing alongside the increasing challenge for medical healthcare systems in managing an ageing population [51]. Much of the key evidence presented from large, landmark randomized controlled trials almost completely excludes (< 1%) patients > 80 years [14, 16, 52, 53]. Thus, there is no firmly established 1L SOC for elderly DLBCL patients.

### Palliative Approaches

The management of elderly DLBCL patients is often complex. Fundamental to holistic therapy is a consideration of the patient’s wishes alongside careful assessment of comorbidities, polypharmacy, frailty, vulnerability to infection and impaired PS, all of which contribute to treatment-related complications. Very frail or particularly elderly patients (i.e. ≥ 85 years) may wish to undergo a palliative-based approach, focusing on quality of life and symptom control. To that end, data supports anthracycline-free approaches (for example, CVP+/−R; cyclophosphamide, vincristine, prednisolone, +/−rituximab or CEOP; cyclophosphamide, etoposide, vincristine and prednisolone). A recent large, retrospective Danish series noted that, independent of comorbidity index (in this instance, Charlson Comorbidity Index (CCI)), those > 85 years had similar OS when receiving CVP+/−R or CEOP+/−R compared with standard R-CHOP or R-CHOEP (R-CHOP plus etoposide). Lower intensity regimens, such as steroids or rituximab monotherapy, offer inferior disease control and subsequently worse survival. However, these options may be entirely appropriate in the very frail or those wishing to avoid well-established chemoimmunotherapy-related toxicities [54].

### Curative Approaches: R-mini-CHOP

A summary of recent, key prospective clinical trials investigating curative regimens in elderly patients are outlined (Table 1**)**. The view that anthracycline-based chemotherapy could not be utilized safely with curative potential in the elderly was challenged by the practice changing LYSA phase II trial [55] which assessed attenuated (or ‘mini’) R-CHOP in 150 patients > 80 years. Patients received 6 cycles of R-mini-CHOP (25 mg/m^2^ doxorubicin, 400 mg/m^2^ cyclophosphamide, 1 mg capped-dose vincristine). The median age was 83 years (range 80–95) and the 2-year PFS was 47% and 2-year OS was 59%. Cumulative incidence of relapse was not initially reported. A plateau emerged on the survival analysis curves suggesting, for the first time in a prospective trial, that patients > 80 years could receive curative anthracycline-based therapy. With longer follow-up (41 months), the 4-year estimated OS was 49.3% and 4-year disease free survival (DFS) was 57.9% [56]. G3-4 neutropenia occurred in 39% and NF in 7%. Notably, there were 12 (8%) deaths from treatment-related toxicity. As a result, a subsequent phase II trial [57] from LYSA introduced pre-phase therapy (vincristine-prednisolone) in an attempt to reduced toxic deaths pre-mini-CHOP. Rather than rituximab, ofatumumab-mini-CHOP was investigated. Ofatumumab is a fully human monoclonal IgG anti-CD20 antibody that targets a unique membrane-proximal epitope of CD20 with increased affinity and a longer dissociation time. The study reported no toxic deaths in 120 patients treated despite the median age being identical to the R-mini-CHOP trial. The 2-year PFS was 57.2%, 2-year OS was 64.7% and 2-year DFS estimate was 66.6%. Seventy-eight percent received G-CSF prophylaxis. G3-4 neutropenia and NF were reported in fewer patients (21% and 1% respectively). Although cross-trial comparisons are challenging, two population characteristics were also similar in terms of age-adjusted IPI, albumin and geriatric assessment. This provides prospective evidence for the value of pre-phase vincristine-prednisolone, with the suggestion that such an approach may reduce subsequent TRM, by establishing early disease control and improving patient PS in a non-toxic fashion. The relative benefit of ofatumumab is debatable and this agent has not replaced rituximab as the antibody of choice.

**Table 1:** Summary of selected prospective front-line trials in elderly patients with DLBCL

Series	Years	*N*	Regimen	Prospective trials	Median age (years)	TRM	PFS	OS	Comments
Peyrade et al. (2011) [55]	2006–2009	150	R-mini-CHOP	Phase II	83	8%	2-year PFS 47%	2-year OS 59% 4-year OS 49.3%	Albumin level at diagnosis was key independent predictor of outcome on multivariable analysis
Fields et al. (2014) [74]	2008–2010	62	R-GCVP	Phase II	76.5	6%*	2-year PFS 49.8%	2-year OS 55.8%	15 cardiac events (including 5 grade 3–4 and 3 deaths
Jung et al. (2015) [102]	2010–2013	51	R-CHOP × 4 plus 4 weekly rituximab	Phase II	76	12%	2-year PFS 63.9%	2-year OS 68.7%	6 deaths on R-CHOP (4 infection)
Park et al. (2016) [103]	2011–2013	23	R-B	Phase II	80	17%	Median PFS 5.4 months	Median OS 10.2 months	52% ECOG PS ≥ 2. Trial terminated for futility at interim analysis
Storti et al. (2018) [104]	2012–2014	49	R-B	Phase II	81	Not reported	Median PFS 10 months 2-year PFS 38%	2-year OS 51%	90 mg/m^2^ every 4 weeks Bendamustine dose
Peyrade et al. (2017) [57]	2010–2011	120	O-mini-CHOP	Phase II	83	0%	2-year PFS 57.2%	2-year OS 64.7%	Pre-phase vincristine-prednisolone
Shen et al. (2018) [76]	2012–2015	61	R-GemOx	Phase II	75	0%	3-year PFS 49%	3-year OS 65%	Similar results in patients ≥ 80 years. All patients with CCI ≥ 3.
Luminari et al. (2018) [75]	2009–2011	50	R-COMP	Phase II	76	Not reported	Median 17 months 3-year PFS 38%	3-year OS 50%	21% cardiac AEs. 12% grade 3–4 AEs. No cardiac related deaths

*TRM*, treatment-related mortality; *OS*, overall survival; *PFS*, progression-free survival; *R-GCVP*, rituximab, gemcitabine, cyclophosphamide, vincristine, prednisolone; *CHOP*, cyclophosphamide, doxorubicin, vincristine, prednisolone; *O*, ofatumumab; *GemOx*, gemcitabine and oxaliplatin; *R-B*, rituximab-bendamustine; *R-COMP*, rituximab, non-pegylated liposomal doxorubicin, cyclophosphamide, vincristine, prednisolone; *ECOG PS*, Eastern Cooperative Oncology Group performance score *3 additional cardiac related deaths

### Pre-Phase and G-CSF Prophylaxis

A recent non-randomized cohort comparison study [58] (pre-phase *n* = 50, no pre-phase *n* = 50 with well-matched baseline characteristics) also showed the potential benefit of pre-phase vincristine-prednisolone. There was a significant improvement in PS with 92% in the pre-phase group having an Eastern Cooperative Oncology Group PS (ECOG PS) 0–1 pre-R-CHOP from only 36% at diagnosis. This resulted in a subsequent reduction in rates of all grade neutropenia, G3-4 neutropenia and NF (pre-phase 16% vs. non-phase 34%; *p* = 0.037).

Pooled clinical trial data from 520 patients over 60 years treated with R-CHOP (*n* = 250) or CHOP (*n* = 270) suggests that the NF risk is highest in cycle 1 (38% of all NF episodes occurred at cycle 1). This phenomenon was particularly apparent on multivariable analysis in patients with a lower baseline haemoglobin (< 12 g/dl) (odds ratio (OR) = 2.2) and > 65 years (OR = 2.6) [59]. Only 2% received primary G-CSF prophylaxis from cycle 1 as these were not allowed per protocol, compared with 22–29% from cycle 5–8. Although historical data is somewhat mixed and not specifically focused on the elderly, studies broadly show that G-CSF reduces neutropenia and infection, enabling retention of relative dose intensity (RDI) without impacting OS [60–62]. It is recommended that primary G-CSF prophylaxis should be used in DLBCL patients ≥ 65 years receiving R-CHOP [63].

### Dose Intensity of Attenuated R-CHOP in the Elderly

In light of above studies, many more patients are receiving attenuated anthracycline-based chemoimmunotherapy as a recognized standard. There remains, however, an open question as to what dose intensity, particularly with reference to doxorubicin and cyclophosphamide, is necessary in often complex elderly patients, in order to optimize efficacy without compromising safety. To date, no randomized trials comparing dose(s) of cyclophosphamide and doxorubicin in elderly patients have been performed.

To this end, there are relatively few retrospective studies that have systematically addressed this question. Small studies have documented, perhaps unsurprisingly, that retaining RDI is broadly important. RDI < 70% was identified as a poor independent prognostic factor in 152 patients (*p* = 0.007) (only 114 were > 60 years) [64]. Reducing cyclophosphamide or doxorubicin to < 90% RDI in cycle 1–2 was associated with inferior outcomes in 140 patients ≥ 70 years in an Israeli study [65].

We [66] and others [67] have previously analysed the impact of planned dose reductions at treatment initiation, i.e. intended dose intensity (IDI) in small series. Although IDI reduction commonly results in a reduced overall RDI, these may be associated with less toxicity and an overall improved or equivalent outcome in elderly patients. Full dose vs attenuated R-CHOP did not improve outcome in 1L patients > 80 years and may in fact worsen survival via increased toxicity.

A recent Danish population-based analysis included 557 DLBCL patients > 75 years treated with R-CHOP (full or attenuated) with available IDI information [54]. IDI < 80% (defined by whether either cyclophosphamide or doxorubicin dose were < 80% in cycle 1) when compared with > 80% were associated with a similar OS in patients > 80 years, suggesting that R-CHOP could be reasonably attenuated in that age. Data here was limited however by the lack of integrated RDI analysis, the relatively large number of unknown causes of death and the consequent lack of assessment of cumulative relapse risk.

Our recent data [68, 69] showed that in a large cohort of 690 consecutive elderly patients of ≥ 70 years treated with full or attenuated R-CHOP, when comparing patients 70–80 years with ≥ 80 years, there was no difference in the cumulative incidence of relapse, when performing a competing risk analysis for non-relapse mortality. When patients ≥ 80 years were separately analysed, there was no clear benefits in terms of relapse rate, PFS and OS to full dose R-CHOP (defined as IDI > 80% of the combination of cyclophosphamide and doxorubicin) compared with R-mini-CHOP (IDI < 80%).

### Options in Anthracycline-Unfit

Doxorubicin is a key component of R-CHOP, but it is associated cardiac toxicity, particularly congestive cardiac failure, with increasing cumulative dose [70]. A large US study showed the risk of cardiomyopathy is worsened in patients with co-existent hypertension [71]. As such, patients with cardiac compromise are typically considered unfit for anthracycline-based, curative chemoimmunotherapy. Gemcitabine is an cytotoxic antimetabolite with clear activity in R/R DLBCL, often in combination with platinum-based treatment and high-dose steroids with no cardiac toxicity [72, 73]. Gemcitabine has been studied as a direct anthracycline substitute (R-GCVP) within R-CHOP. R-GCVP was assessed in a phase II trial [74] of 62 patients who either had a documented left ventricular ejection fraction (LVEF) ≤ 50%, as assessed by transthoracic echocardiogram or multigated acquisition nuclear medicine scan; or cardiac comorbidities (hypertension, IHD or diabetes) which precluded anthracycline use. The median age was 76.5 years. 43.5% had a reduced LVEF ≤ 50% and 56.5% had comorbid cardiac risk factors. Response rate was 61.3% with (29% CR). Two-year PFS was 49.8% and 2-year OS was 55.8%. Fifteen cardiac events were noted (G1-2 (*n* = 7); G3-4 (*n* = 5)) including 3 deaths, reflecting the nature of the population studied. Forty-seven percent developed G3-4 neutropenia and G ≥ 3 infection was reported in 28%. These results suggested that selected elderly patients with cardiac comorbidities receive curative non-anthracycline-based therapy with manageable toxicity, although thorough pre-treatment cardiovascular assessment and medical optimisation is mandatory. Outcomes of 50 patients with cardiac comorbidity receiving liposomal doxorubicin as substitute for standard doxorubicin (R-COMP) were recently published and showed slightly inferior results (3-year PFS 38%, 3-year OS 50%) to R-GCVP in a similar age group. A similar number of cardiac events (12% G3-4)) were reported including 3 concerning cases of LVEF reduction ≥ 20% from baseline, although no directly related cardiac deaths were reported [75].

Gemcitabine has also been combined with oxaliplatin and rituximab (R-Gem-Ox every 14 days up to 6 cycles) in a recent phase II trial [76] of patients ≥ 70 years or 60–69 years with an ECOG PS ≥ 2. The median age of the 60 patients enrolled was 75 years and 45% had an ECOG PS ≥ 2. G3-4 neutropenia occurred in 15% and no treatment-related deaths occurred. The response rate was 75% and 3-year PFS was 49% and 3-year OS was 65%. Survival for patients ≥ 80 years was strikingly similar to the whole cohort (3-year OS 67% and 3-year PFS 49%). Neither ECOG PS ≥ 2 or CCI ≥ 5 were predictive for worse PFS or OS on univariable analysis. IPI 3-5 was associated with worse outcome for PFS and OS on univariable and multivariable analysis (multivariable analysis: PFS HR 2.6 *p* = 0.024; OS HR 4.4 *p* = 0.004). Overall, these results suggest that gemcitabine-based chemoimmunotherapy can provide cure in a tolerable fashion in ~ 50% of elderly DLBCL patients, including those with cardiac and other comorbidities. On this basis, a randomized trial (R-GemOx vs R-mini-CHOP) comparing safety and efficacy in elderly DLBCL patients is recruiting (NCT02767674).

**Fig. 1:**
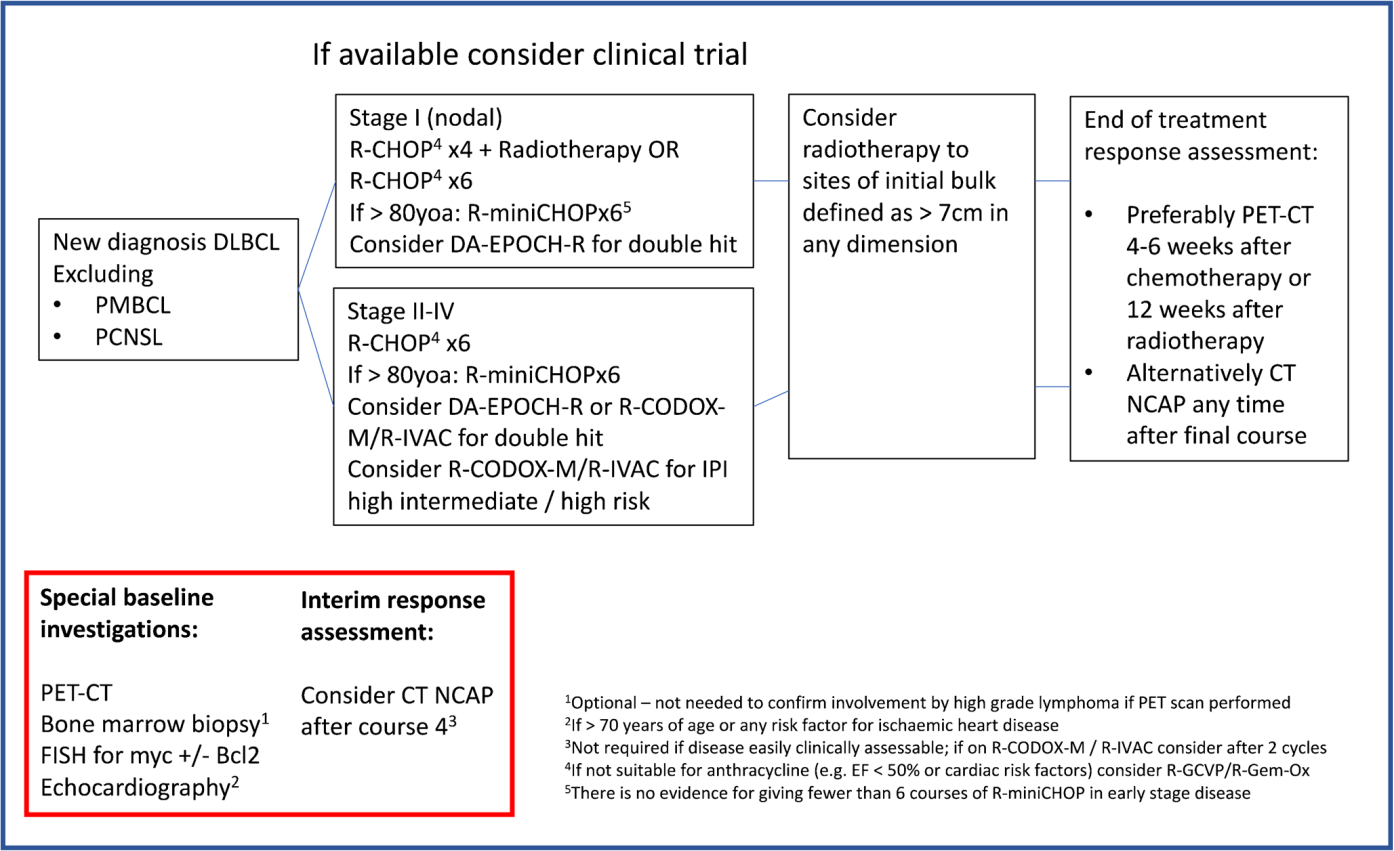
Suggested pathway for front-line management of DLBCL. DLBCL, diffuse large B cell lymphoma; PMBCL, Primary mediastinal B cell lymphoma; PCNSL, Primary central nervous system lymphoma; R-CHOP, rituximab, cyclophosphamide, vincristine, prednisolone; CT NCAP, Computed tomography neck, chest, abdomen and pelvis; DA-EPOCH-R, dose-adjusted etoposide plus rituximab, cyclophosphamide, vincristine, prednisolone; R-GCVP, rituximab, gemcitabine, cyclophosphamide, vincristine, prednisolone; PET-CT, ^18^F-fluorodeoxyglucose enhanced positron emission tomography with concurrent low dose computed tomography; EF, left ventricular ejection fraction

In summary, available data suggests that R-CHOP remains the SOC in elderly DLBCL, although the age and frailty that should guide dose attenuation remain to be fully defined. Pre-phase vincristine-prednisolone should be strongly considered where the baseline PS may risk a higher TRM and morbidity and primary G-CSF prophylaxis is recommended in all ≥ 65 years. In patients ≥ 80 years, there is accumulating evidence for the lack of benefit of escalation of doxorubicin and cyclophosphamide dose beyond ‘mini-CHOP’ RDI. There is no gold standard, prospectively validated, comorbidity scoring system to guide therapeutic choice in elderly DLBCL, although patients with cardiac comorbidity can be successfully treated with gemcitabine-based chemoimmunotherapy following careful optimisation. Our standard approach to 1L management of DLBCL, including elderly patients, is outlined in Fig. 1.

## PMBCL

PMBCL is a rare, aggressive, thymic B cell derived lymphoma, predominantly affecting adolescents and young adults (AYA) [77–79]. Features overlap with both classical Hodgkin lymphoma (cHL) and other B cell NHL subtypes, thus making accurate diagnosis and optimal therapy challenging. Significant advances in molecular diagnostics recognize PMBCL as a distinct entity, with unique clinicopathological features [3, 4, 80, 81]. Although this has enhanced diagnostic accuracy, therapeutic advances have been limited, especially within the context of young patients at significant risk of long-term complications of chemoimmunotherapy and radiotherapy [82], especially cardiac toxicity [83–86] and secondary malignancy [87, 88]. There remains a paucity of prospective data to define the optimal 1L approach [89].

### R-CHOP or DA-EPOCH-R?

Data from the MiNT trial [15] which included 22% PMBCL patients, confirmed a EFS, PFS, and OS benefit of rituximab alongside CHOP-like regimens [90]. This supported previous retrospective data, establishing R-chemotherapy as the SOC in PMBCL [91–93]. Commonly used regimens are R-CHOP and DA-EPOCH-R, however, no randomized trials comparing them in PMBCL.

1L R-CHOP induces sustained remission in ~ 80% of PMBCL patients [90, 94]. The results of the IELSG-26 trial established the prognostic utility of PET-CT-based response, and within the previously described PETAL trial, for the small number PMBCL patients randomized based on interim PET-CT response, intensification failed to improve outcomes [45, 95].

The largest reported series of R-CHOP outcomes in PMBCL was a subgroup analysis of NCRI phase III trial comparing R-CHOP14 vs. R-CHOP21. Despite a trend toward improved survival with R-CHOP-14, this sub-analysis failed to reveal any significant difference between regimens. At a median follow-up over 7 years, 50 patients experienced a 5-year PFS and OS of 79.8% and 83.8% respectively [96]. These results are comparable with the PMBCL subgroup within MiNT [97].

The majority of R-CHOP trials in PMBCL have included involved site radiotherapy (ISRT) consolidation. To date, there is no robust data to support the safety of abandoning ISRT post-R-CHOP [98]. Whether or not ISRT can be omitted safely in patients achieving PET-CT defined complete metabolic response (CMR) with R-CHOP will hopefully be addressed by the forthcoming prospective IESLG-37 study (NCT01599559).

In those wishing to avoid radiotherapy, DA-EPOCH-R has been the most widely studied regimen. Dunleavy reported an EFS and OS of 93% and 97% respectively at a median follow-up in excess of 5 years in 51 patients. Although achieving a CMR rate of 96% without ISRT, there was considerable myelotoxicity and infection [99]. Furthermore, the same response rate was not observed in a large, multicentre, retrospective analysis of adults and children treated with DA-EPOCH-R. This study reported a 3-year EFS and OS of 85.9% and 95.4% respectively. End-of-treatment PET-CT CMR was 75% and ISRT rate was 14.9% at a median dose of 36 Gy [100]. The previously mentioned phase III CALBG/Alliance 53,003 trial included a small cohort of PMBCL patients (6.9% R-CHOP and 5.8% DA-EPOCH-R). As such, the superiority of DA-EPOCH-R over R-CHOP + ISRT in PMBCL is not established [78]. R-CHOP + ISRT remains a recommended SOC, with DA-EPOCH-R as an alternative in cases where the benefit of ISRT is outweighed by risk [91]. With respect to DA-EPOCH-R, given the observation of ongoing PET-CT responses post-therapy [99], scanning should be performed ≥ 6 weeks following completion. Although it is safe to omit ISRT in patients achieving end-of-treatment CMR (Deauville 1-3) post DA-EPOCH-R induction, it may still be considered. For young females, the International Late Effects of Childhood Cancer Guideline Harmonization Group recommends annual breast cancer surveillance with either mammography or breast MRI (or combination), for female childhood, adolescent and young adult cancer survivors treated with ≥ 20 Gy chest radiation. Breast cancer surveillance is recommended from age 25 years or ≥ 8 years from radiation, for at least up to 50 years [101].

## Conclusion

Despite our increased understanding of the pathobiology and mechanisms of therapy resistance of DLBCL and PMBCL, both diseases represent an area of unmet need with respect to enhancing the efficacy of 1L chemoimmunotherapy strategies especially in vulnerable patient populations. Our progress in understanding has been coupled with the development of targeted novel agents and technologies. The challenge now is to harness these advances for the benefit of patients without compromising their long-term safety and quality of life.
